# Risk factors of delayed hemorrhage after LigaSure hemorrhoidectomy

**DOI:** 10.1186/s12893-022-01802-w

**Published:** 2022-10-13

**Authors:** Kung-Chuan Cheng, Ling-Chiao Song, Kuen-Lin Wu, Hong-Hwa Chen, Ko-Chao Lee

**Affiliations:** 1 Division of Colorectal Surgery, Department of Surgery, Kaohsiung Chang Gung Memorial Hospital, Chang Gung University College of Medicine, 833 Kaohsiung, Taiwan; 2 Division of Colon & Rectal Surgery, Department of Surgery, E-DA Hospital, I-Shou University, 824 Kaohsiung, Taiwan

**Keywords:** Hemorrhoidectomy, LigaSure, Delayed post-hemorrhoidectomy bleeding

## Abstract

**Background:**

As one of the most popular methods for treating hemorrhoidal diseases, hemorrhoidectomy with LigaSure devices has been proven to have less postoperative pain and has gained in popularity among surgeons. However, our previous study found higher incidence of delayed post-hemorrhoidectomy bleeding (DPHB) in patients who underwent LigaSure hemorrhoidectomy compared to those who underwent the traditional Ferguson’s method. This follow-up study aimed to reveal the relationship between DPHB and the surgeon’s experience.

**Methods:**

This retrospective study included 437 consecutive patients with symptomatic grade II to IV hemorrhoids who received hemorrhoidectomy by LigaSure devices from March 2009 to December 2017. Twenty-two patients who experienced DPHB were analyzed to identify risk factors. Cumulative incidence of DPHB were calculated and visualized to assess the improvement of DPHB rate by time.

**Results:**

All operations were performed by a single surgeon. The most common postoperative complication was constipation, followed by urinary retention. DPHB developed in 22 patients (5%). Multivariate analysis showed that the male sex was an independent risk factor for DPHB in patients who underwent hemorrhoidectomy with LigaSure devices. The cumulative incidence was initially higher (about 10%) in the earlier cases and stabilized at around 5% with more cases. The change in cumulative incidence indicated a lower complication rate as the surgeon’s experience increased.

**Conclusion:**

Male sex is an independent risk factor for DHBP. The risk of DPHB is higher in patients undergoing hemorrhoidectomy with LigaSure in a surgeon’s earlier cases, and decreases to a rate similar to that for the traditional hemorrhoidectomy once the surgeon becomes more familiar with the procedure and postoperative care.

**Supplementary Information:**

The online version contains supplementary material available at 10.1186/s12893-022-01802-w.

## Background

About 40% of adults suffer from hemorrhoidal disease, making it one of the commonest disease in the general population [1]. Goligher classification is a traditional and widely-used tool to evaluate the severity of hemorrhoidal disease[2], and surgical excision is the first choice of treatment for grade III and IV or recurrent hemorrhoids[3]. Open hemorrhoidectomy (Milligan and Morgan procedure) and closed hemorrhoidectomy (Ferguson procedure) are the most commonly used surgical techniques for the excision of hemorrhoids and remain the gold standard of surgical treatment [4]. However, these procedures are associated with complications such as pain and postoperative bleeding. Therefore, colorectal surgeons are dedicated to search for a hemorrhoidectomy technique that is more effective and has less complications.

The LigaSure vessel sealing system (Valleylab, Boulder, CO, USA) is a hemostatic device designed primarily for use in abdominal surgeries. Using a combination of pressure and electrical energy, it ensures complete coagulation of vessel with minimal surrounding thermal spread and limited tissue scarring [5]. Several randomized trials have compared LigaSure hemorrhoidectomy with conventional hemorrhoidectomy [6–10]. The LigaSure method significantly reduces postoperative pain by causing less tissue injury and scarring, and this has led to its increasing popularity as a choice for hemorrhoidectomy.

Delayed post-hemorrhoidectomy bleeding (DPHB) is a rare but serious complication after hemorrhoidectomy with a reported incidence of about 0.9-10% [11]. Past studies found that the risk factors of DPHB included surgical procedure, sex, infection, defecation with excessive straining, laxative usage, and number of piles [12, 13]. Previously, we reported higher risk of DPHB with LigaSure hemorrhoidectomy compared with the conventional Ferguson’s Method [12]. In this study, we retrospectively analyzed the risk factors of DPHB following LigaSure hemorrhoidectomy performed by a single surgeon. Furthermore, we longitudinally analyzed the DPHB rate and revealed the trend by time.

## Methods

### Study population

We initially identified 513 consecutive patients with symptomatic grade II to IV hemorrhoids who underwent elective hemorrhoidectomy performed by a single colorectal surgeon at the Kaohsiung Chang Gung Memorial Hospital between March 2009 to December 2017. Patients who received procedures other than LigaSure hemorrhoidectomy were excluded (n = 76), and a total of 437 Patients were included in this study. There were no pregnant women included in this cohort. Patients on antithrombotic or antiplatelet agents were asked to discontinue their medication 7 days prior to surgery. Patients were admitted to the Surgery ward one day before, or in the morning of the procedure and were discharged the day after operation unless postoperative complications developed.

During the operation, the patient was placed in the modified Sims’ position. The procedure started with an incision at the base of hemorrhoids, followed by submucosal dissection to lift the hemorrhoidal tissue off from sphincters using monopolar devices. Then the jaw of the Ligasure was applied on the pedicles of hemorrhoids, and the device was activated to ligate the vessels. Two different LigaSure devices (LF4200 and LF1212A) were used in this study. The details of surgical procedures are described in our previous study [12].

In the event of postoperative bleeding, our initial procedures included an initial rectal irrigation to determine whether there was acute bleeding. If irrigation showed continuous fresh blood indicating active bleeding of the operation site, an operation was arranged and carried out in the operation room where the surgeon would achieve hemostasis. If irrigation showed blood clots and the fluid became clearer indicating no active bleeding, then the patient was treated with bowel rest and intravenous fluid support. For this group of patients, we took into consideration the patient’s baseline health status to determine whether hospitalization was required. For example, elderly patients or patients with comorbidities were admitted for close observation.

This study was approved by the institutional review board of the Chang Gung Medical Foundation (approval number: 202200266B0). Since this was a retrospective analysis in which patients treated with clinical routines were enrolled, the requirement for participants’ informed consent was waived. All study methods were performed in accordance with the relevant guidelines and regulations of IRB of Chang Gung Memorial Hospital.

### Definitions

Anemia was defined by a serum hemoglobin level lower than 13.5 g/dL in males and 12 g/dL in females. We defined constipation as defecation characterized by infrequent stools, difficult stool passage, or both. The finding of constipation was based on patients’ self-reported difficulty in passing stool. This definition included incomplete evacuation and preoperative laxative usage. Stool impaction was defined as development of solid, immobile feces in the rectum causing discomfort, an urge to defecate, easy wound bleeding, and anal contraction. We treated cases of constipation with impaction with oil retention and cleansing enemas to allow discharge of the stool retained at the end of the rectum. Urinary retention was defined as dysuria with need for indwelling urinary catheter. DPHB was defined as a patient who presented to the emergency department with more than a bowel (200ml) of anal bleeding after discharge and within 30 days after operation.

### Statistical analysis

Continuous variables were expressed as medians and interquartile ranges, and categorical variables were expressed as frequencies and percentages. The logistic regression model estimated OR (odds ratio) and 95% CI (confidence interval) to quantify the strength of association between different factors and postoperative bleeding. Factors with *p* values < 0.05 were subsequently used to establish the multivariable regression model. *p* values < 0.05 were considered statistically significant. All statistical analyses were two-sided and performed with Statistical Package for the Social Sciences Version 25.0 software (SPSS, Chicago, IL, USA). Cumulative incidence of DPHB was calculated and visualized using Microsoft Excel 2010 (Microsoft, Washington, USA).

## Results

### Patients’ characteristics

A total of 437 patients from March 2009 to December 2017 were included. In our study cohort, the median age was 50 years old, and 44.9% of patients were male. Anemia was noted in 30.1% of patients preoperatively. 265 patients underwent hemorrhoidectomies by LF4200, and 39.8% of patients received suture of pedicles. The most common postoperative complication in our study cohort was constipation (n = 74, 16.9%), followed by urinary retention (n = 62, 14.2%). DPHB developed in 22 patients (5%). Further details of patient characteristics and operative procedures are listed in Table 1. Postoperative outcomes were listed in Table 2. The entire study cohort were showed in Supplementary Table 1.

**Table 1:** Baseline characteristics

Characteristics	n
Age (median(IQR))	50(40–59)
Sex (%)	
Male	196 (44.9)
Female	241 (55.1)
Wounds status (%)	
< 3 wounds	54 (12.4)
≥ 3 wounds	383 (87.6)
Suturing of Pedicles (%)	
Yes	174 (39.8)
No	263 (60.2)
LigaSure Device (%)	
LF4200	265 (60.6)
LF1212A	172 (39.4)
Vital signs (median(IQR))	
SBP (median(IQR))	120(110–132)
DBP (median(IQR))	73(68–80)
Pulse rate (median(IQR))	72(64–78)
Hemoglobin (median(IQR))	13.3(12.1–14.3)
Platelet (median(IQR))	244(211–283)
Comorbidities (%)	
Diabetes	18 (4.1)
Hypertension	57 (13.0)
Coronary artery disease	8 (1.8)
End-stage renal disease	2 (0.5)
Anemia	131 (30)
Stroke	1 (0.2)
Chronic hepatitis	16 (3.7%)
Asthma/COPD	3 (0.7)
Preoperative anticoagulants	5 (1.1

**Table 2:** Incidence of postoperative complications

Complications	n
DPHB (%)	22 (5)
Urinary retention (%)	62 (14.2)
Constipation (%)	74 (16.9)
Stool impaction (%)	10 (2.3)

*　DPHB: Delayed post-hemorrhoidectomy bleeding

### DPHB in LigaSure hemorrhoidectomy

There were 22 cases of DPHB. Univariate analysis showed that male, end-stage renal disease, and postoperative stool impaction were risk factors for DPHB. Results from the multivariate analysis showed that the male sex (OR 3.26, 95% CI 1.24–8.61, *p* = 0.017) and stool impaction (OR 5.23, 95% CI 1-27.413, *p* = 0.05) were independent risk factors for DPHB as shown in Table 3.

**Table 3:** Odds ratio on univariate and multivariate logistic regression analysis

	Univariate Analysis			Multivariate Analysis	
**Variables**	**OR (95% CI)**	***p*** **value**		**OR (95% CI)**	***p*** **value**
Age	1.01 (0.97–1.04)				
Male	3.48 (1.34–9.08)	0.011		3.26 (1.23–8.61)	0.017
≥ 3 wounds	3.07 (0.41–23.33)	0.277			
Suturing of pedicles	0.93 (0.59–1.45)	0.733			
LigaSure device(LF1212A)	1.07 (0.45–2.56)	0.879			
Vital signs					
Systolic blood pressure	1.01 (0.99–1.04)	0.261			
Diastolic blood pressure	1.02 (0.98–1.06)	0.304			
Pulse rate	1.02 (0.97–1.07)	0.489			
Hemoglobin	1.00 (0.95–1.05)	0.559			
Platelet	1.00 (0.99-1.00)	0.482			
Comorbidities					
Hypertension	1.06 (0.30–3.69)	0.932			
Coronary artery disease	2.76 (0.33–23.49)	0.352			
End-stage renal disease	19.71 (1.19–326.25)	0.037		12.87(0.76-217.02)	0.076
Anemia	1.15 (0.44–3.00)	0.776			
Chronic hepatitis	2.86 (0.61–13.47)	0.183			
Preoperative anticoagulants	4.89 (0.52–45.72)	0.164			
Outcomes					
Urinary retention	1.05 (0.30–3.66)	0.939			
Constipation	1.31 (0.38–4.54)	0.673			
Stool impaction	5.05 (1.00-25.35)	0.049		5.23 (1.00-27.41)	0.050

In the 22 patients with DPHB, the median interval between hemorrhoidectomy and bleeding was 7 days. The median hemoglobin on the day of DPHB occurrence was 12.1 g/dL. Hypotension was noted in 18.2% (n = 4) of patients. 18 out of 22 patients required hospitalization for management of the DPHB, and 12 patients required surgical hemostatic intervention by either ligation or electrocauterization. 2 patients developed recurrent bleeding, and both were admitted for hospitalization on the first bleeding event, during which one patient underwent surgical hemostasis. The other patient also underwent surgical hemostasis eventually. The 4 patients who did not require hospitalization were relatively young and healthy, and were discharged from the emergency department after a couple of hours of observation since they had stable vital signs and there were no evidence of acute hemorrhage. Details are listed in Table 4.

**Table 4:** Characteristics and outcome of DPHB patients

Characteristics	*n*
Days from hemorrhoidectomy to bleeding (median (INR))	7 (4–9)
Hemoglobin (median (INR))	12.1 (11.3–13.3)
Blood transfusion in ED (%)	5 (22.7)
Hypotension (%)	4 (18.2)
Admission (%)	18 (81.8)
length of hospital stay (median (INR))	3.5 (2–5)
Management	
Observation (%)	4 (18.2)
Rectal irrigation / compression (%)	6 (27.3)
Surgical hemostasis(%)	12 (54.5)
Urinary retention(%)	8 (36.4)
Recurrent bleeding (%)	2 (9.1)

### Cumulative incidence of DPHB

**Fig. 1:**
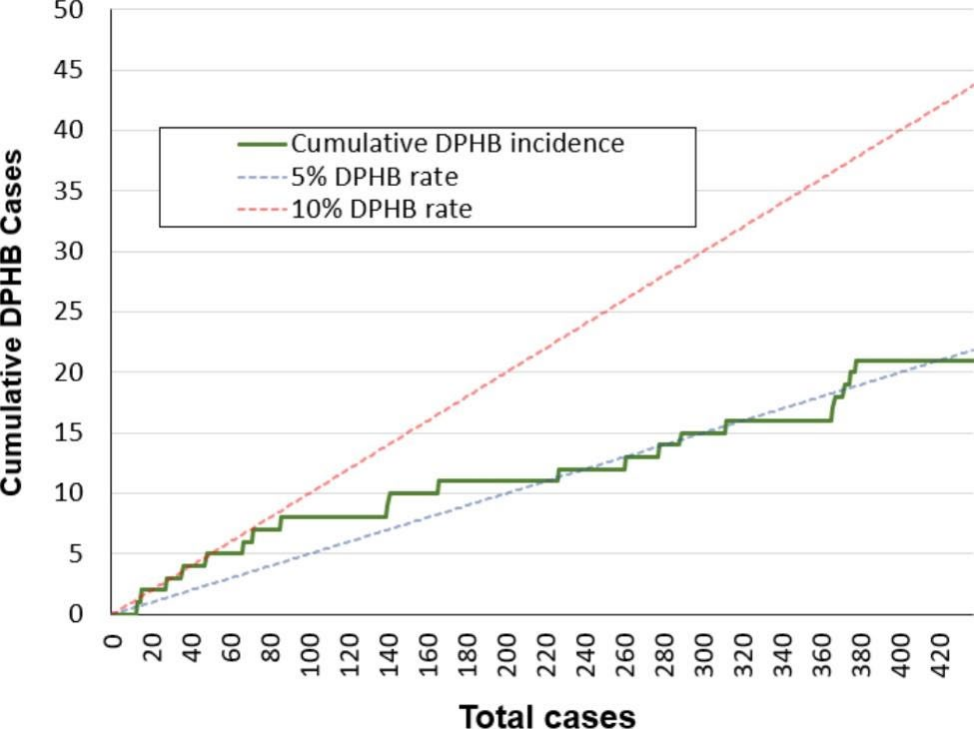
Cumulative DPHB incidence

We calculated the cumulative sum of DPHB rate and visualized it (Fig. 1). The initial DPHB rate started at around 10% and gradually decreased after 87 cases. The cumulative rate stabilized at around 5% after 193 cases, and this is similar to the DPHB rate for the traditional Ferguson method reported in our previous study[12]. We must mention that our post-hemorrhoidectomy patient care protocol has changed since January 2010. The new postoperative care plan focuses more on informing patients to avoid heavy-lifting and intensive exercises within the 2 weeks after operation. This may be a confounder to the change in DPHB rate by time.

## Discussions

Hemorrhoidectomy is the most effective and definitive treatment for symptomatic hemorrhoids. Though conventional hemorrhoidectomy has mostly been used, pain-related complications have often caused prolonged hospital stays. Besides, conventional procedures were also associated with postoperative complications such as tissue trauma, bleeding, and anal stricture[14].

Nowadays, the LigaSure device, as an improved version of bipolar diathermy, are being used more frequently in many surgical procedures including hemorrhoidectomy. Recent studies have reported that patients who received the LigaSure procedure experienced lower postoperative pain, reduced tissue damage, and more rapid wound healing compared to conventional hemorrhoidectomy [15–17], and this corresponds with our clinical observation. In our experience, patients who underwent LigasSure hemorrhoidectomy presented with less postoperative pain than those who underwent the Ferguson procedures, especially during the week after operation. Consequently, procedures with the LigaSure device have led to earlier hospital discharge and return to normal activity.

Besides post-operative pain, DPHB is a rare but serious complication after hemorrhoidectomy. Previous studies have demonstrated that the male sex was an independent risk factor for DPHB [18, 19], and this study also found similar result.In addition to patients’ characteristics, type of surgical procedure may be another risk factors for DPHB. Several studies have been conducted to compare LigaSure hemorrhoidectomy and traditional hemorrhoidectomy, and results regarding DPHB have been inconsistent. Our previous study found that LigaSure hemorrhoidectomy had significantly higher DPHB rate when compared to Ferguson hemorrhoidectomy [12]. On the other hand, in a randomized prospective study, Tshijanu et al. compared LigaSure hemorrhoidectomy with the classical Milligan-Morgan Procedure. They reported less postoperative pain and recurrence rate in the LigaSure group. There was no significant difference between the two procedural groups regarding other postoperative events, such as bleeding, fever, urinary retention, fecal incontinence, anal stenosis [20]. Similarly, Khanna et al. compared LigaSure hemorrhoidectomy with conventional ‘closed’ Ferguson’ s hemorrhoidectomy, and the DPHB rates were also similar between the two groups [10].

This study showed that the male gender and stool impaction may be potential risk factors for DPHB, and this is consistent with our previous report. However, in our previous report, patients who received LigaSure hemorrhoidectomy seemed to have higher incidence of DPHB compared with those who received the traditional Ferguson procedure [12]. This report is a longitudinal research of patients who underwent Ligasure hemorrhoidectomy performed by a single surgeon, and the goal is to investigate the correlations between LigaSure hemorrhoidectomy and DPHB from another aspect. Our data showed that DPHB rate was indeed higher in the surgeon’s earlier cases. However, the cumulative bleeding rate gradually decreased after 87 cases and eventually stabilized at about 5% after 193 cases, as shown in Fig. 1. This is close to the bleeding rate found in patients who underwent traditional procedures in our previous study.

For patients who receive the traditional Ferguson procedure, the postoperative pain limits their activity. On the other hand, patients who receive LigaSure hemorrhoidectomy tend to be more active after the operation due to less postoperative pain. We assume that DPHB is related to the intensity of patients’ daily activity after the operation. This may explain the initially higher cumulative DPHB rate in the patients who underwent LigaSure hemorrhoidectomy. As the surgeon gained experience, became more familiar with the energy devices, and put more emphasis on postoperative care and informing patients to avoid heavy-lifting and intensive exercises after hemorrhoidectomy, the cumulative rate of DPHB gradually decreased to a rate similar to that for the Ferguson method, implying that the use of LigaSure device is not a risk of DPHB for experienced surgeons. Further prospective study is needed to support this hypothesis.

Through surgical excision remains the mainstream treatment of hemorrhoidal disease, there are several non-excisional managements which are less invasive, such as rubber band ligation, sclerotherapy, stapled hemorrhoidopexy, and transanal hemorrhoidal dearterialization[3]. For example, Gallo et al. reported satisfactory results for the treatment of grade II hemorrhoidal disease using 3% polidocanol foam for sclerotherapy[21]. Moreover, P. Giordano and E. Schembari demonstrated that transanal hemorrhoidal dearterialization can be used in more advanced hemorrhoidal disease with slight modification[22]. However, in Taiwan, surgical excision remains the most dominant procedure for hemorrhoidal disease due to policies of the National Health Insurance.

In this study, we provided the postoperative results of LigaSure hemorrhoidectomies performed by the same surgeon. In this way, we eliminated the confounder of different surgeons as mentioned in another report [18], and showed the trend for change in cumulative incidence of DPHB as the surgeon became more experienced. To the best of our knowledge, this is the largest dataset reported on LigaSure hemorrhoidectomies performed by a single surgeon, and the first study to use a cumulative chart to visualize the change in the cumulative incidence of DPHB. This may provide some practical information for the training of young surgeons. Nonetheless, this study has some limitations. First, this is a retrospective and non-randomized analysis, and therefore selection bias may exist. Second, the case number of DPHB is small (n = 22), which may limit the study’s power to show other independent risk factors. It is important to validate our findings in large, multi-center, prospective trials.

## Conclusion

The male sex is an independent risk factor for DPHB in patients undergoing hemorrhoidectomy with the LigaSure device. In addition, the risk of DPHB may be higher in a surgeon’s earlier cases and may gradually decrease once the surgeon becomes more familiar with the procedure and the corresponding postoperative care. The cumulative bleeding risk of LigaSure hemorrhoidectomy eventually stabilizes to a rate similar to that for the traditional hemorrhoidectomy.

## Electronic supplementary material

Below is the link to the electronic supplementary material.

Supplementary Table 1. Entire study cohort

## Data Availability

All data analyzed during this study are included in this published article and its supplementary information files.

## Abbreviations

DPHB: Delayed post-hemorrhoidectomy bleeding.
OR: odds ratio.
CI: confidence interval.
